# Attenuation of diabetic cardiomyopathy by relying on kirenol to suppress inflammation in a diabetic rat model

**DOI:** 10.1111/jcmm.14638

**Published:** 2019-09-29

**Authors:** Bin Wu, Xue‐Yuan Huang, Le Li, Xiao‐Hang Fan, Peng‐Cheng Li, Chuan‐Qi Huang, Juan Xiao, Rong Gui, Shun Wang

**Affiliations:** ^1^ Laboratory of Platelet and Endothelium Biology, Department of Transfusion Medicine, Wuhan Hospital of Traditional Chinese and Western Medicine, Tongji Medical College Huazhong University of Science and Technology Wuhan China; ^2^ Department of Transfusion Medicine, the Third Xiangya Hospital Central South University Changsha China; ^3^ Department of Physiology and Pharmacology, Medical College Hubei University of Arts and Science Xiangyang China; ^4^ Department of Cardiology, the Third Xiangya Hospital Central South University Changsha China; ^5^ Department of Pathophysiology, Tongji Medical College Huazhong University of Science and Technology Wuhan China; ^6^ Department of Pharmacy, Wuhan Hospital of Traditional Chinese and Western Medicine, Tongji Medical College Huazhong University of Science and Technology Wuhan China; ^7^ Department of Immunology, Medical College Hubei University of Arts and Science Xiangyang China

**Keywords:** cardiac dysfunction, diabetic cardiomyopathy, fibrosis, inflammation, myocardial remodelling

## Abstract

Diabetic cardiomyopathy is characterized by diabetes‐induced myocardial abnormalities, accompanied by inflammatory response and alterations in inflammation‐related signalling pathways. Kirenol, isolated from *Herba Siegesbeckiae*, has potent anti‐inflammatory properties. In this study, we aimed to investigate the cardioprotective effect of kirenol against DCM and underlying the potential mechanisms in a type 2 diabetes mellitus model. Kirenol treatment significantly decreased high glucose‐induced cardiofibroblasts proliferation and increased the cardiomyocytes viability, prevented the loss of mitochondrial membrane potential and further attenuated cardiomyocytes apoptosis, accompanied by a reduction in apoptosis‐related protein expression. Kirenol gavage could affect the expression of pro‐inflammatory cytokines in a dose‐dependent manner but not lower lipid profiles, and only decrease fasting plasma glucose, fasting plasma insulin and mean HbA1c levels in high‐dose kirenol‐treated group at some time‐points. Left ventricular dysfunction, hypertrophy, fibrosis and cell apoptosis, as structural and functional abnormalities, were ameliorated by kirenol administration. Moreover, in diabetic hearts, oral kirenol significantly attenuated activation of mitogen‐activated protein kinase subfamily and nuclear translocation of NF‐κB and Smad2/3 and decreased phosphorylation of IκBα and both fibrosis‐related and apoptosis‐related proteins. In an Electrophoretic mobility shift assay, the binding activities of NF‐κB, Smad3/4, SP1 and AP‐1 in the nucleus of diabetic myocardium were significantly down‐regulated by kirenol treatment. Additionally, high dose significantly enhanced myocardial Akt phosphorylation without intraperitoneal injection of insulin. Kirenol may have potent cardioprotective effects on treating for the established diabetic cardiomyopathy, which involves the inhibition of inflammation and fibrosis‐related signalling pathways and is independent of lowering hyperglycaemia, hyperinsulinemia and lipid profiles.

## 1 INTRODUCTION

Diabetic cardiomyopathy (DCM), which is generally recognized as abnormalities in myocardial structure and function, including hypertrophy, fibrosis, apoptosis, dilatation and systolic dysfunction, occurs in the diabetic population in the absence of coronary atherosclerosis, valvular heart disease, hypertension and other congenital heart disease.^1^, ^2^ Interestingly, accumulating evidence from recent studies suggests that several molecular mechanisms involved in the progression of left ventricular (LV) remodelling converge towards inflammation and fibrosis‐related signalling pathways including nuclear factor‐κB (NF‐κB), the mitogen‐activated protein kinase (MAPK) and transforming growth factor‐β (TGF‐β)/Smad pathways.^3^, ^4^, ^5^ Moreover, insulin resistance and mitochondrial dysfunction generally occur during the progression of myocardial remodelling and are also responsible for promoting diabetic myocardial inflammation.^6^, ^7^

Currently, several classes of oral antidiabetic drugs, such as metformin, sulfonylureas, meglitinides, pioglitazone, α‐glucosidase inhibitors, DPP‐4 inhibitors, GLP‐1 receptor agonists and SGLT2 inhibitors, are available for type 2 diabetes mellitus (T2DM) patients.^8^ However, these medications cannot effectively reverse the pathological changes in DCM patients.^9^ Therefore, accumulating evidence from preclinical studies suggests that using natural herbal extracts to treat diabetes‐related complications may satisfy clinical physicians and scientists to a certain extent due to their multiple treatment targets.^10^ Kirenol is the major active diterpenoid component extracted from *Herba Siegesbeckiae* and can inhibit activation of NF‐κB signalling and the expression of pro‐inflammatory cytokines (eg IL‐1β) due to its anti‐inflammatory responses in collagen‐induced arthritis (CIA).^11^ It has also been reported to possess immunomodulatory, anti‐tumour and anti‐UV‐induced photoageing properties.^12^, ^13^, ^14^ Kirenol can down‐regulate the expression levels of key adipogenic transcription factors and inhibit lipogenesis of 3T3‐L1 adipocytes in vitro.^15^ However, the evidence of kirenol as a promising therapy for DCM has not yet been investigated, and the anti‐DCM molecular mechanisms of kirenol remain unclear.

The Goto‐Kakizaki (GK) rat is a T2DM rodent model that displays defective β‐cell mass, mild fasting hyperglycaemia and insulin resistance, and impaired glucose‐mediated insulin secretion; thus, diabetes mellitus in these rats is similar to T2DM in humans.^16^, ^17^ Herein, we hypothesized that kirenol may protect against DCM by attenuating cardiac remodelling via inhibition of both inflammatory and fibrotic signalling pathways. This study evaluated (a) whether kirenol could prevent cardiofibroblasts (CFs) proliferation and decrease fibrosis‐related protein expression in HG‐induced CFs; (b) whether kirenol could inhibit HG‐induced cardiomyocytes (CMs) apoptosis and mitochondrial dysfunction via inactivation of Bax and caspase‐3 in vitro; (c) whether long‐term oral administration of kirenol could ameliorate the progression of cardiac remodelling and improve cardiac function in GK rats; and (d) whether the potential molecular mechanisms of kirenol were related to inhibition of the NF‐κB, TGF‐β/Smad and MAPK signalling pathways and activation of Akt.

## 2 MATERIALS AND METHODS

### 2.1 Cell proliferation and viability assay

**Figure 1:**
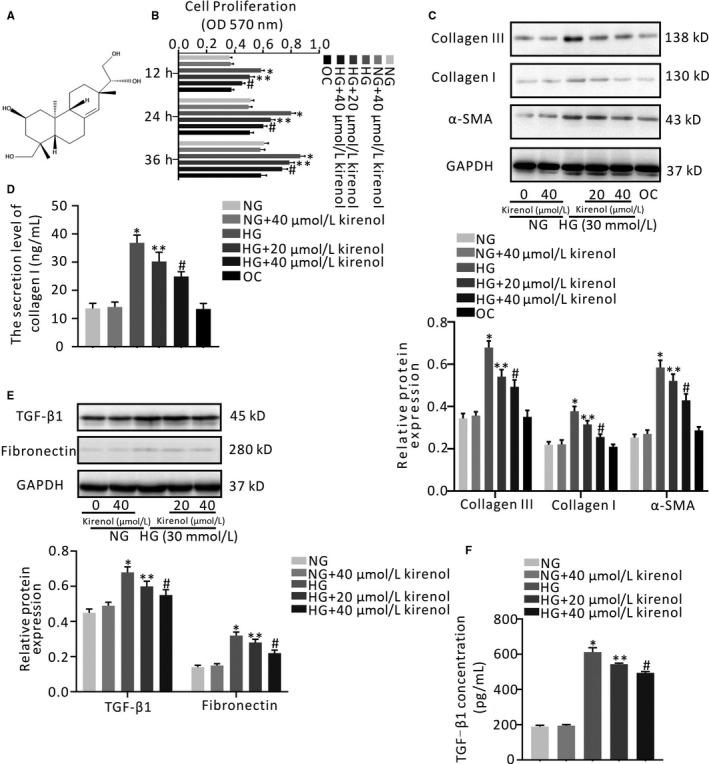
Effect of kirenol on HG‐induced proliferation of cardiofibroblasts and fibrosis‐related protein expression in cardiofibroblasts. A, Chemical structure of kirenol (B) CFs were incubated with NG medium, HG medium, HG medium combined with different concentrations of kirenol, NG medium with 40 μmol/L kirenol (NG with kirenol control) and NG medium with mannose (OC), at the indicated time‐points (12, 24 and 36 h). Cell proliferation was assessed by MTT assay, C, The protein expressions of collagen III, collagen I and α‐SMA were analysed by Western blotting. GAPDH expression was served as an internal control. The target protein levels were normalized to GAPDH, D, The accumulation level of collagen I via myofibroblasts secretion in supernatant was measured by ELISA assay, E, CFs were treated with NG medium, NG medium with 40 μmol/L kirenol, HG medium, kirenol (20 or 40 μmol/L) in the presence of HG for 36 h, respectively. The expression of TGF‐β1 and fibronectin in CFs, as well as (F) secretory TGF‐β1 in CFs supernatant were determined by Western blotting. The data shown are mean ± SD of three independent experiments. *P < .05 vs NG; **P < .05; ^#^ *P* < .05 vs HG

The primary CMs and CFs were separately collected from the heart of neonatal Wistar rats and cultured as previously described.^18^ Cell proliferation was determined by MTT assay (Sigma‐Aldrich, Shanghai, China) according to the manufacturer's instructions. Briefly, after starvation in serum‐free medium (Gibco) for 24 hours, CFs were incubated in DMEM‐F12 (Gibco) containing 5.5 mmol/L d‐glucose (normal d‐glucose, NG; Sigma‐Aldrich), 30 mmol/L d‐glucose (high d‐glucose, HG; Sigma‐Aldrich) and 5.5 mmol/L d‐glucose plus 27.5 mmol/L mannose (osmotic control, OC; Sigma‐Aldrich). Kirenol (Desite Biotech; Figure 1A) dissolved in distilled water was added to the medium and maintained at final concentrations of 20 and 40 μmol/L when CFs were exposed to NG or HG, respectively. The kirenol treatment was performed for 12, 24 or 36 hours at various concentrations. Neonatal myoblasts were randomly divided into six groups after incubation in serum‐free M199 medium (Gibco) for 48 hours and exposed to the following different treatments: (a) NG control (5.5 mmol/L d‐glucose), (b) NG control with 40 μmol/L kirenol, (c) HG control (30 mmol/L d‐glucose; HG), (d) HG plus 20 μmol/L kirenol and (e) HG plus 40 μmol/L kirenol. Cell Counting Kit‐8 (CCK‐8; Sigma‐Aldrich) was measured in all groups according to the manufacturer's instructions. Finally, the absorbance of MTT assay and CCK‐8 assay was measured at 570nm and 490nm, respectively, using a Microplate Reader (Thermo Scientific).

### 2.2 Apoptosis analysis by flow cytometry

Mitochondrial membrane potential (MMP) was assessed with the fluorescent probe JC‐1 (Beyotime Biotechnology) according to the manufacturer's instructions. The JC‐1 monomers/aggregates ratio represented early cell apoptosis through the MMP. By calculating the ratio of JC‐1 monomers to aggregates, the extent of MMP decline is represented. The apoptotic rates of CMs were determined with flow cytometry using an Annexin V‐FITC/propidium iodide (PI) staining kit (Beyotime Biotechnology, Shanghai, China) according to the manufacturer's instructions. Stained cells in different groups were observed and analysed with a FACS Calibur™ flow cytometer (BD Sciences) with FlowJo X software.

### 2.3 Animal experimental protocols

Male GK rats (8 weeks of age) together with age‐matched male Wistar rats were purchased from Slack Jinda Animal Technology Co. Ltd. All animals were housed at constant room temperature (23 ± 1°C) under 12 hours light and 12 hours dark cycles and allowed access to chow food and water ad libitum. The experimental protocols involving all rats in the present study were approved by the Institutional Animal Care and Use Committee of Xiangya Medical College of Central South University, and the rats were kept according to the institutional ethical guidelines of Central South University.

The GK rat is relatively lean and displays several representative features of T2DM as early as four months of age (16 weeks of age).^16^ ALL rats were fed until they were 16 weeks old. Sixteen‐week‐old rats were randomly assigned into four groups: a Wistar control group (n = 8, CT), a GK control group (n = 8, GKCT), a low‐dose kirenol‐treated GK group (n = 8, LDKT) and a high‐dose kirenol‐treated GK group (n = 8, HDKT). The rats in both the CT group and GKCT group were administered equal quantities of saline by gavage, while the diabetic rats were treated with 0.5 and 2 mg/kg of kirenol daily by gavage for 8 weeks. The fasting plasma glucose (FPG) levels, the fasting plasma insulin levels and bodyweights of all rats were measured once a week. At the end of the observation, animals were weighed, anesthetized and examined for LV function by haemodynamic measurements. After haemodynamic measurements were obtained, four rats were randomly selected from each group to receive intraperitoneal injections of insulin. Next, all rats were sacrificed. Whole blood was collected and then separated in a tube with sodium heparin (MedChemExpress), and the separated plasma was further analysed for biochemical parameters. The heart tissue was then removed and rinsed, the LVs were isolated, dried, immediately frozen in liquid nitrogen and stored at −80°C until use. The timeline of the experimental procedures is shown in Figure S1.

### 2.4 Haemodynamic measurements

In vivo pressure‐volume (PV) loop analysis of the LV was used to acquire and evaluate cardiac function as previously described.^2^, ^19^ Briefly, haemodynamic measurements were performed using a Millar catheter (SPR‐838; Millar). The haemodynamic parameters were recorded and analysed using a Powerlab system (ADInstruments).

### 2.5 Determination of biochemical parameters

FPG was measured by test strips of a OneTouch glucometer (Johnson and Johnson). According to the manufacturer's instructions, glycosylated haemoglobin (HbA1c) and Lipid profiles including total cholesterol (TC), triglycerides (TG), low‐density lipoprotein (LDL) cholesterol and high‐density lipoprotein (HDL) cholesterol in plasma were assessed with commercial kits (Jiancheng Bioengineering Institute).

### 2.6 Determination of insulin, collagen I, TGF‐β1, TNF‐α, IL‐1β and IL‐6 Levels

Collagen I and TGF‐β1 secretion levels in CFs supernatants were measured using an ELISA kit (R&D System). LV tissues were homogenized in pre‐chilled PBS (pH 7.4), centrifuged at 12 000 × *g* for 20 minutes at 4°C and used for evaluating these pro‐inflammatory and pro‐fibrotic cytokines. The levels of TNF‐α, IL‐1β and IL‐6 in plasma were determined using commercially available ELISA kits (Bosterbio). The ELISA kit of Rat insulin was purchased from Invitrogen for determining insulin in plasma (Shanghai, China).

### 2.7 Cardiac histological examination

After sacrifice, myocardial samples were prepared for histological examination. Hearts isolated from all rats were fixed with 4% paraformaldehyde (Servicebio), embedded in paraffin and sliced into 4‐ or 10‐μm thick sections. These sections were stained with haematoxylin and eosin (H&E; Servicebio) to measure the cardiac cross‐sectional area (CSA) and with Masson's trichrome (Servicebio) to detect collagen deposition in the heart. For analyses, stained slides were observed under a Zeiss photomicroscope (Shanghai, China), and images were quantified by at least two investigators in a blinded manner conducting with Image‐Pro Plus 6.0 software (Media Cybermetics).

### 2.8 TUNEL assay

Terminal deoxynucleotidyl transferase‐mediated dUTP nick‐end labelling (TUNEL) staining was used to confirm apoptosis. Apoptotic nuclei were examined using a TdT In Situ Apoptosis Detection Kit (DAB; R&D Systems) according to the manufacturer's instructions. The percentage of apoptotic cells was calculated as the ratio of the number of TUNEL‐positive cells to the total number of cells.

### 2.9 Protein extraction

According to standard protocols, a cold radioimmunoprecipitation assay (RIPA) lysis buffer (Abcam) containing 100X protease and phosphatase inhibitors (Transgene) was used to obtain total protein extracts of cell lysates and heart tissue samples. The nuclear and cytoplasmic fractions were obtained using the NE‐PER^®^ Nuclear and Cytoplasmic Extraction Kit and Halt™ Protease Inhibitor Cocktail (Thermo Scientific) following the manufacturer's procedures. The protein concentrations of the lysates were determined using the BCA Protein Assay Kit (Bosterbio).

### 2.10 Western blot analysis

Total, nuclear and cytoplasmic extracts (40 μg of protein per lane) were subjected to electrophoresis on 8% or 10% SDS‐PAGE, and the gel was transferred onto polyvinylidene fluoride (PVDF) membranes (ROCHE). The washed PVDF membranes were blocked with PBS containing 5% BSA at room temperature (RT) and then incubated overnight at 4°C with primary antibodies. After further washing, the membranes were exposed to the corresponding horseradish peroxidase‐conjugated secondary antibodies for 2 hours at RT before detection using an enhanced chemiluminescence kit (Thermo Scientific). The primary antibodies used were collagen I (rabbit, 1:1000 dilution, Abcam), collagen III (rabbit, 1:1000 dilution, Abcam), TGF‐β1 (rabbit, 1:1000 dilution, Cell Signaling Technology, Inc), α‐SMA (rabbit, 1:1000 dilution, Cell Signaling Technology, Inc), connective tissue growth factor (CTGF; rabbit, 1:1000 dilution, Cell Signaling Technology, Inc), fibronectin (rabbit, 1:1000 dilution, Abcam), Bcl‐2 (rabbit, 1:1000 dilution, Abcam), Bax (rabbit, 1:1000 dilution, Cell Signaling Technology, Inc), caspase‐3 (rabbit, 1:1000 dilution, Cell Signaling Technology, Inc), atrial natriuretic peptide (ANP; mouse, 1:1000 dilution, Santa Cruz Biotechnology), brain natriuretic peptide (BNP; mouse, 1:1000 dilution, Santa Cruz Biotechnology), phospho‐JNK(Thr183/Tyr185; rabbit, 1:1000 dilution, Cell Signaling Technology, Inc), JNK1/2 (rabbit, 1:1000 dilution, Cell Signaling Technology, Inc), phospho‐p44/42 MAPK (ERK1/2; rabbit, 1:1000 dilution, Cell Signaling Technology, Inc), p44/42 MAPK (ERK1/2; rabbit, 1:1000 dilution, Cell Signaling Technology, Inc), phospho‐p38MAPK (thr180/tyr182; rabbit, 1:1000 dilution, Cell Signaling Technology, Inc), p38MAPK (α/β; rabbit, 1:1000 dilution, Cell Signaling Technology, Inc), phospho‐Akt (Ser473; rabbit, 1:1000 dilution, Cell Signaling Technology, Inc), Akt, Smad2/3 (rabbit, 1:1000 dilution, Cell Signaling Technology, Inc), phosho‐IκBα (rabbit, 1:1000 dilution, Cell Signaling Technology, Inc), IκBα (rabbit, 1:1000 dilution, Cell Signaling Technology, Inc), NF‐κB (p65; rabbit, 1:1000 dilution, Cell Signaling Technology, Inc), PCNA (mouse, 1:1000 dilution, OriGene), β‐tubulin (mouse, 1:1000 dilution, OriGene), β‐tubulin (mouse, 1:1000 dilution, OriGene) and GAPDH (mouse, 1:1000 dilution, OriGene). The secondary antibodies used were anti‐rabbit IgG (H + L; Goat, 1:5000 dilution, Cell Signaling Technology, Inc) and anti‐mouse IgG (H + L; Goat, 1:5000 dilution, Santa Cruz Biotechnology).

### 2.11 Electrophoretic mobility shift assay (EMSA)

The oligonucleotides were synthesized by Sangon Biotech. Double‐stranded DNA probes were 3′ end‐labelled with biotin using a Biotin 3′ End DNA Labelling Kit (Thermo Scientific). The sequences of the probes are listed in Table S1. Nucleoproteins were incubated with the probes and Poly (dI.dC) for 20 minutes at RT before being resolved on a 6% polyacrylamide gel in 0.5 × Tris‐borate buffer, electrotransferred onto a 0.45‐μm nylon membrane (Biodyne™ B; Thermo) at 100 V for 45 min at 4°C, and then crosslinked to the nylon membrane with an 254nm UV Lamp. Bands were detected with the National Institutes of Health (NIH) ImageJ software.

### 2.12 Statistical analysis

The results are presented as the means ± SD Statistical analysis was performed using the SPSS statistical package (SPSS, version 16.0). Differences among the groups of animals were tested by one‐way ANOVA and Bonferroni's multiple comparison post hoc analysis. Data were considered statistically significant for *P* < .05.

## 3 RESULTS

### 3.1 Kirenol prevents HG‐induced CFs proliferation

As shown in Figure 1B, at the indicated time‐points, HG treatment significantly promoted the proliferation of CFs in a time‐dependent manner compared with NG treatment (*P* < .001). However, the proliferation of CFs gradually decreased after supplementation with kirenol at either 20 or 40 μmol/L (Figure 1B, *P* < .01). In addition, both of OC treatment and NG with 40 μmol/L kirenol treatment had no significant effect on the proliferation of CFs (Figure 1B, *P* > .05).

### 3.2 Effect of kirenol on collagen production and fibrosis‐related markers expression in vitro

To evaluate whether various concentrations of kirenol had inhibitory effects on collagen synthesis and fibrosis‐related markers expression, we treated CFs with kirenol (20 and 40 μmol/L) under HG conditions for 36 hours. As shown in Figure 1C, application of kirenol in a concentration‐dependent manner prevented collagen I and III up‐regulation mediated by HG (*P* < .05, Figure 1C). In addition, α‐SMA expression was also significantly decreased by kirenol treatment in a concentration‐dependent manner (*P* < .05, Figure 1C). Furthermore, there was no significant difference in the expression of collagen III, collagen I or α‐SMA among the OC group, the NG group and the NG with 40 μmol/L kirenol group (Figure 1C). Subsequently, at the indicated time‐point, compared with NG treatment, a significant increase in the secretion level of collagen I was observed for CFs exposed to HG treatment, and type I collagen levels were decreased by kirenol treatment in a concentration‐dependent manner (*P* < .05, Figure 1D). A similar result was also found in that OC treatment neither increased nor decreased the secretion of type I collagen compared with NG treatment (Figure 1D). Similar to the above result, there was no significant difference in the secretion of type I collagen between the NG group and the NG with 40 μmol/L kirenol group (Figure 1D).

Since OC treatment had no obvious effect on CFs proliferation, collagen synthesis or α‐SMA expression of CFs, OC treatment as one of the control treatments was not considered in our subsequent experiments. Western blotting indicated that treatment with HG significantly increased the expression of TGF‐β1 and fibronectin in CF lysates compared with NG treatment, whereas kirenol treatment significantly reduced the expression of TGF‐β1 and fibronectin in CFs under HG conditions in a concentration‐dependent manner (*P* < .05, Figure 1E). Similarly, HG also significantly stimulated the expression of TGF‐β1 in CF supernatants (*P* < .05, Figure 1E). Moreover, the CFs TGF‐β1 secretion induced by HG was markedly decreased by kirenol treatment in a concentration‐dependent manner (*P* < .05, Figure 1F). Meanwhile, it was found that NG with 40 μmol/L kirenol treatment did not significantly enhance the expression of TGF‐β1 and fibronectin in CFs and the expression of TGF‐β1 in CF supernatants compared with NG treatment. The data in the present study suggest that kirenol intervention blocked collagen synthesis and fibrosis‐related proteins expression induced by HG in vitro.

### 3.3 Effect of kirenol on CMs viability change and CMs death

**Figure 2:**
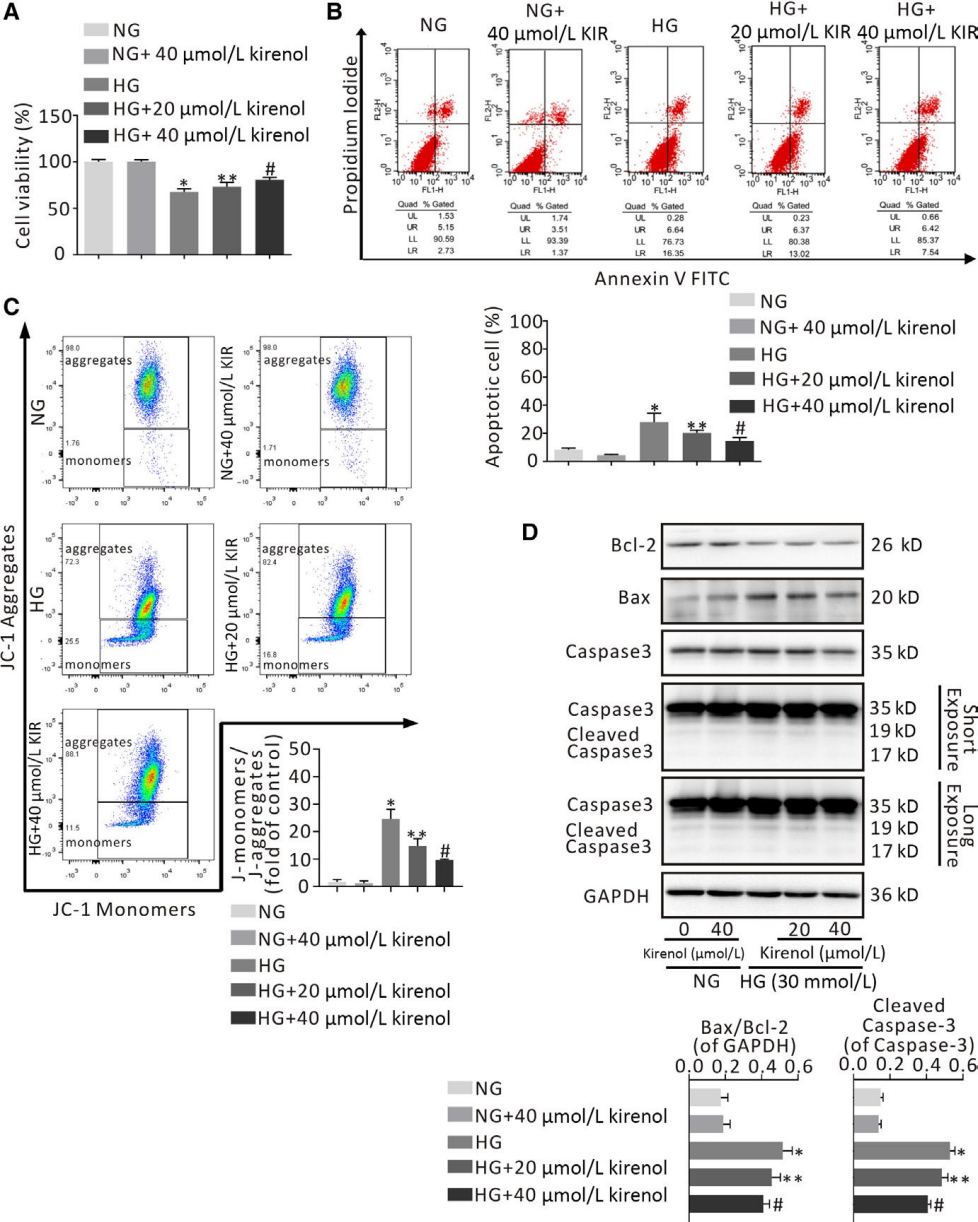
Effects of kirenol treatment on cardiomyocytes viability and apoptosis in the presence of HG. A, CCK‐8 assay was performed to assess the cell viability of HG‐induced CMs treated with various concentrations of kirenol (20 and 40 μmol/L) for 36 h, B, Representative diagram analysis of Annexin V‐FITC/PI staining. Percentage of apoptotic CMs were calculated in the right of lower and upper quadrants. Lower region and upper region represent fluorescence intensities of JC‐1 monomers and JC‐1 aggregates, respectively. The JC‐1 monomers/aggregates ratio (fold of control) exhibits early cell apoptosis through the MMP, C, Kirenol treatment concentration‐dependently decreased the ratio of Bax/Bcl‐2 and down‐regulated the protein expression of cleaved Caspase‐3 in HG‐induced CMs. *P < .001 vs NG; **P < .01; ^#^ *P* < .01 vs HG

To address the protective effects of kirenol in CMs, we investigated the CMsviability change and the apoptotic rates of HG‐exposed CMs determined by CCK‐8 assay and an Annexin V‐FITC/PI flow cytometry assay, respectively, as shown in Figure 2A,B. CCK‐8 assay indicated that there was no obvious difference in CMs viability between NG treatment and NG with 40 μmol/L kirenol treatment (Figure 2A). CMs viability was significantly decreased in the presence of HG, whereas kirenol treatment significantly and concentration‐dependently enhanced the viability of CMs incubated with HG (*P* < .05, Figure 2A). Moreover, there was a significant dose‐dependent reduction in the rate of CMs apoptosis in the kirenol treatment group compared with the HG treatment group (*P* < .05, Figure 2B).

Loss of MMP is an indicator of mitochondrial dysfunction in early apoptotic cells. Therefore, we evaluated the effects of two concentrations of kirenol on MMP in HG‐exposed CMs by flow cytometry of JC‐1 staining, and the results are shown in Figure 2C. A significant increase in MMP levels and a considerably lower ratio of JC‐1 monomers and aggregates occurred in HG‐exposed CMs treated with kirenol in a concentration‐dependent manner, which can be observed in the representative photographs (*P* < .001, Figure 2C). It was also observed that NG with 40 μmol/L kirenol treatment neither up‐regulated the rate of CMs apoptosis nor reduced MMP levels compared with NG treatment (Figure 2B,C).

Subsequently, we explored the effect of kirenol on the levels of apoptosis‐related proteins by immunoblotting. Consistent with the results of previous experiments, the ratio of Bax/Bcl‐2 was significantly higher in HG‐exposed CMs than in NG‐treated CMs and was dramatically decreased by both moderate and high concentrations of kirenol (20 and 40 μmol/L; *P* < .001, Figure 2D). In addition, cleaved caspase‐3 expression was significantly down‐regulated by kirenol treatment in a concentration‐dependent manner under HG conditions (*P* < .01, Figure 2D). Similar to the results of OC treatment on CFs, NG with 40 μmol/L kirenol treatment did not significantly up‐regulate the ratio of Bax/Bcl‐2 and cleave caspase‐3 expression in CMs compared with NG treatment (Figure 2D).

### 3.4 Effect of kirenol on bodyweight, FPG and biochemical parameters in GK rats

As presented in Figure S2, there was no difference in bodyweight in all the GK groups after 8 weeks of kirenol treatment. No significant differences in the mean FPG and fasting plasma insulin were observed between the GKCT and LDKT groups (*P* > .05). After high‐dose kirenol treatment for 8 weeks, the diabetic rats in the HDKT group showed considerably lower concentrations of FPG and fasting plasma insulin at the indicated time‐points than those in the GKCT group, with a statistically significant difference (*P* < .01, Figure S2). At the end‐point of the kirenol administration experiment, only GK rats in the HDKT group displayed a significant decrease in the mean HbA1c level (*P* < .05; Table S2). Lipid profiles including TG, TC, HDL‐cholesterol and LDL‐cholesterol levels were not affected by kirenol administration.

### 3.5 Kirenol gavage decreased plasma levels of TNF‐α, IL‐1 β and IL‐6

Long‐term diabetes significantly increased plasma levels of TNF‐α, IL‐1 β and IL‐6 in diabetic rats without daily oral kirenol administration. However, oral administration of kirenol for 8 weeks dose‐dependently down‐regulated increasing plasma TNF‐α, IL‐1 β and IL‐6 levels (Table S3).

### 3.6 Kirenol treatment improves diabetic cardiac function

**Figure 3:**
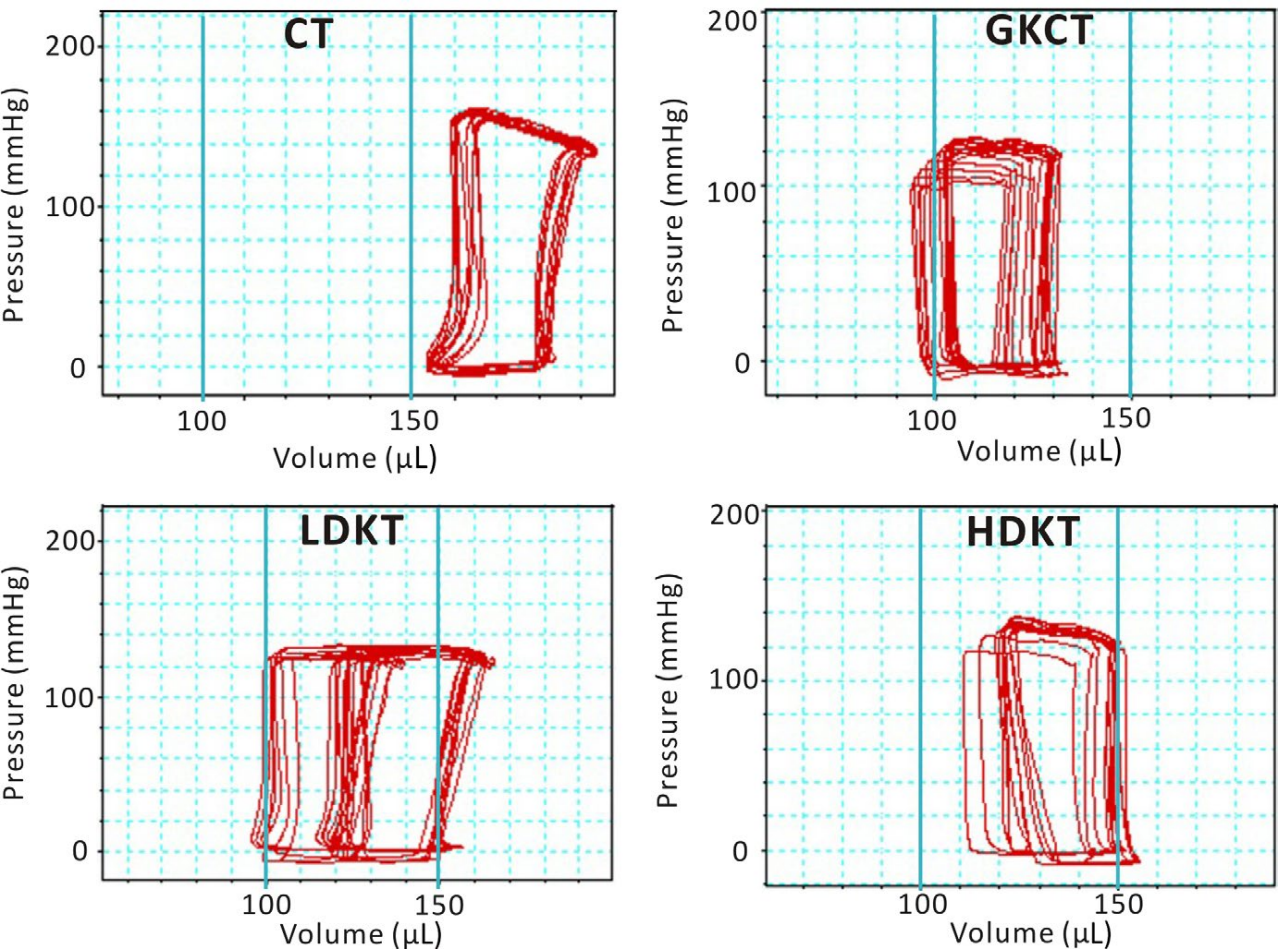
Representative Photomicrographs of pressure‐volume loops. The figure shows daily oral administration of kirenol (0.5 and 2.0 mg/kg/d) significantly improves left ventricular function of the diabetic animals

At the end of the animal study, haemodynamic measurements were used to evaluate cardiac function in all the groups. The haemodynamic data indicated that the heart rate (HR), end‐systolic pressure (Pes), end‐diastolic pressure (Ped), maximal rates of rise of ventricular pressure (d*p*/d*t*_max_) and maximal rates of decline of ventricular pressure (d*p*/d*t*_min_) in the GKCT group were considerably lower than those in the CT group (*P* < .01, Table S4). However, as shown in Table S4, eight weeks of kirenol treatment increased HR and improved cardiac systolic and diastolic function in a dose‐dependent manner (*P* < .01). Representative photomicrographs of the PV loops are illustrated in Figure 3. Oral administration of two concentrations of kirenol over 8 weeks shifted the PV loops to the right compared with diabetic PV loops in Figure 3. The results suggest that kirenol gavage could attenuate these impairments in cardiac function.

### 3.7 Kirenol treatment ameliorated myocardial remodelling

**Figure 4:**
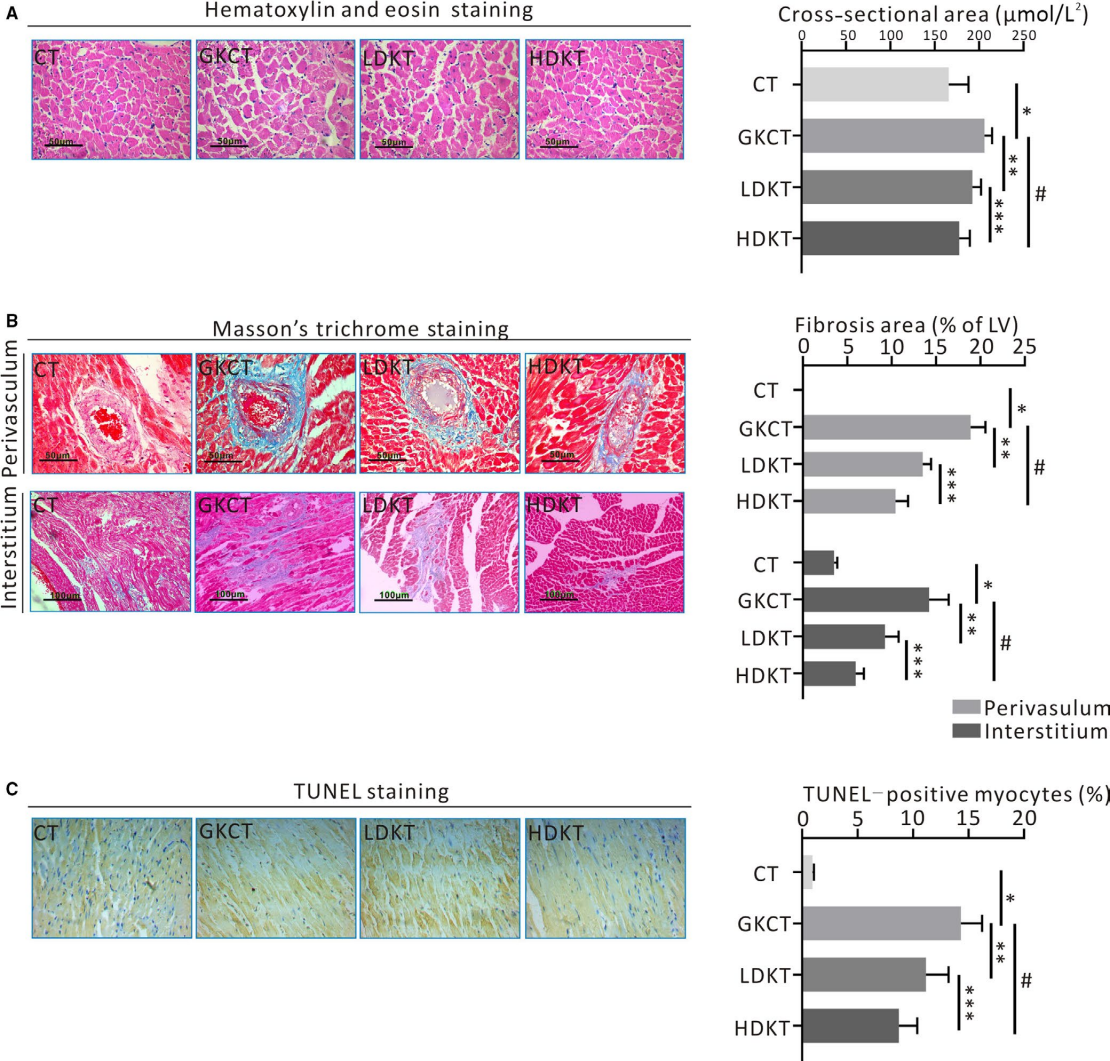
Kirenol attenuated left ventricular remodelling in diabetic GK rats. A, Representative haematoxylin and eosin staining indicated cardiomyocyte cross‐sectional area (magnification ×400; scale bar: 50 μm), and quantitative analysis ofCSA, B, Representative photomicrographs of Masson trichromatic staining for cardiac fibrosis analysis (magnification ×200 or ×400; scale bar: 50 or 100 μm). Cardiac muscle fibres were dyed red; cardiac collagen fibres were dyed blue. Quantitative analysis of the fibrotic area revealed the ratio of the fibrotic area to the area of perivasculum and interstitum, C, TUNEL assay for myocardial apoptosis (magnification ×400): nucleus with dark brown‐stained on behalf of apoptosis. *P < .01 vs CT; **P < .01; ^#^ *P* < .01 vs GKCT; ***P < .01 vs LDKT

Kirenol treatment for 8 weeks could suppress myocardial atrophy induced by diabetes in a dose‐dependent manner (*P* < .01, Figure 4A). Moreover, both ANP and BNP levels were dramatically increased in the myocardium of GK rats, while daily oral administration of kirenol significantly alleviated the expression levels of ANP and BNP in diabetic myocardium (Figure S3A).

Subsequently, the appearance of collagen deposition or fibrosis induced by T2DM was revealed by Masson's trichrome staining. GK rats that did not receive kirenol gavage showed significant interstitial and perivascular fibrosis induced by diabetes (Figure 4B). However, kirenol gavage dose‐dependently prevented LV fibrosis in GK rats compared with the diabetic rats in the GKCT group (Figure 4B). In addition, we explored whether kirenol had an effect on the expression of fibrosis‐related proteins in the LV myocardium of the experimental animals. First, the protein expression of α‐SMA was significantly elevated in GK rats and attenuated with kirenol treatment in a dose‐dependent manner (Figure S3B). Next, we observed that collagen I, collagen III, fibronectin and CTGF proteins, and α‐SMA were similarly increased in diabetic rats, while the expression levels of these proteins were dramatically decreased in GK rats in the presence of kirenol administration in a dose‐dependent manner (Figure S3B). Moreover, daily oral administration of kirenol significantly reduced the expression of myocardial TGF‐β1 in treated rats compared with those in the GKCT group in a dose‐dependent manner (Figure S3B).

**Figure 5:**
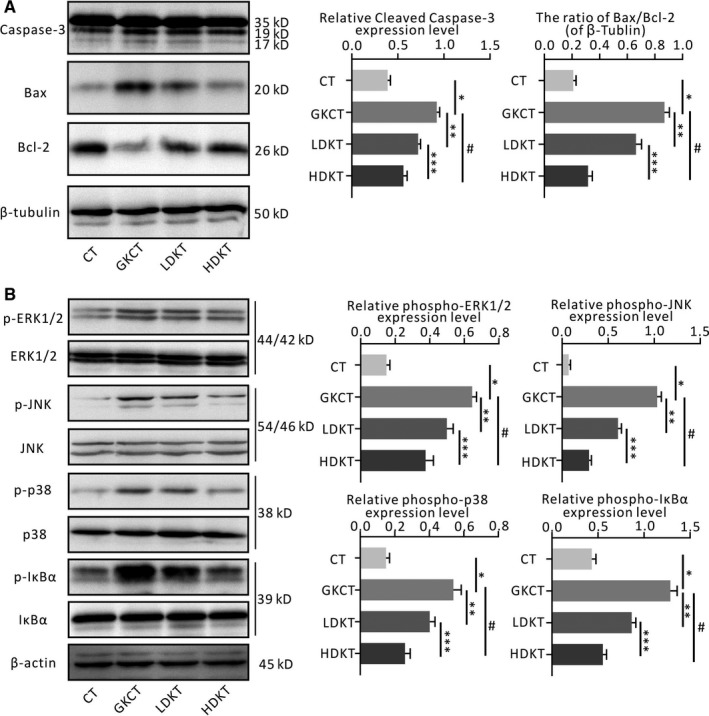
Kirenol ameliorated diabetes induced the apoptotic protein expression and inflammatory signalling pathways activation in myocardium of the GK rats. A, Cleaved Caspase‐3, Bax and Bcl‐2 protein expression detected by Western blotting, B, Western blotting was performed for detecting total and phosphorylated levels of ERK1/2, JNK, p38MAPK and IκBα, as well as β‐tubulin or β‐actin in heart. *P < .01 vs CT; **P < .01; ^#^ *P* < .01 vs GKCT; ***P < .01 vs LDKT

A TUNEL assay was performed to determine CMs apoptosis in rat LVs among different groups. As shown in Figure 4C, various doses of kirenol administration gradually decreased the apoptosis index of CMs induced by diabetes (Figure 4C). Consistently, immunoblotting further indicated that kirenol also dose‐dependently attenuated diabetes‐induced cleaved caspase‐3 and Bax expression and enhanced Bcl expression (Figure 5A).

### 3.8 Kirenol regulates phosphorylation of MAPK and IκBα in the diabetic myocardium

To further investigate the potential mechanism by which kirenol treatment inhibits cardiac remodelling, the MAPK and IκBα signalling pathways were evaluated by Western blotting. In the MAPK signalling pathway, as shown in Figure 5B, the phosphorylation levels of ERK1/2, JNK and p38 that were activated by diabetes were increased in the GKCT group, while the expression of phospho‐ERK1/2, phospho‐JNK and phospho‐p38 were down‐regulated by kirenol treatment in a dose‐dependent manner. Moreover, when we detected phosphorylated IκBα protein levels in the LV, phosphorylated IκBα expression exhibited a significant increase in the diabetic hearts of GK rats but was notably reduced following the treatment of diabetic rats with kirenol in a concentration‐dependent fashion (Figure 5B).

### 3.9 Kirenol enhances Akt phosphorylation in diabetic hearts

As indicated in Figure S4, we found that kirenol treatment at either a low dosage (0.5 mg/kg/d) or a high dosage (2.0 mg/kg/d) significantly reversed the insulin‐induced Akt phosphorylation levels in diabetic hearts. In addition, kirenol also up‐regulated myocardial phosho‐Akt levels in GK rats without intraperitoneal injection of insulin, although the phosphorylation levels were still far below the insulin‐induced levels (Figure S4). Thus, these data demonstrate a profound effect of kirenol on Akt activation and insulin signal transduction.

### 3.10 Daily oral administration of kirenol attenuates Smad2/3 and NF‐κB translocation and the binding activity of NF‐κB, Smad3/4, Sp‐1 and AP1 in diabetic rat myocardium

**Figure 6:**
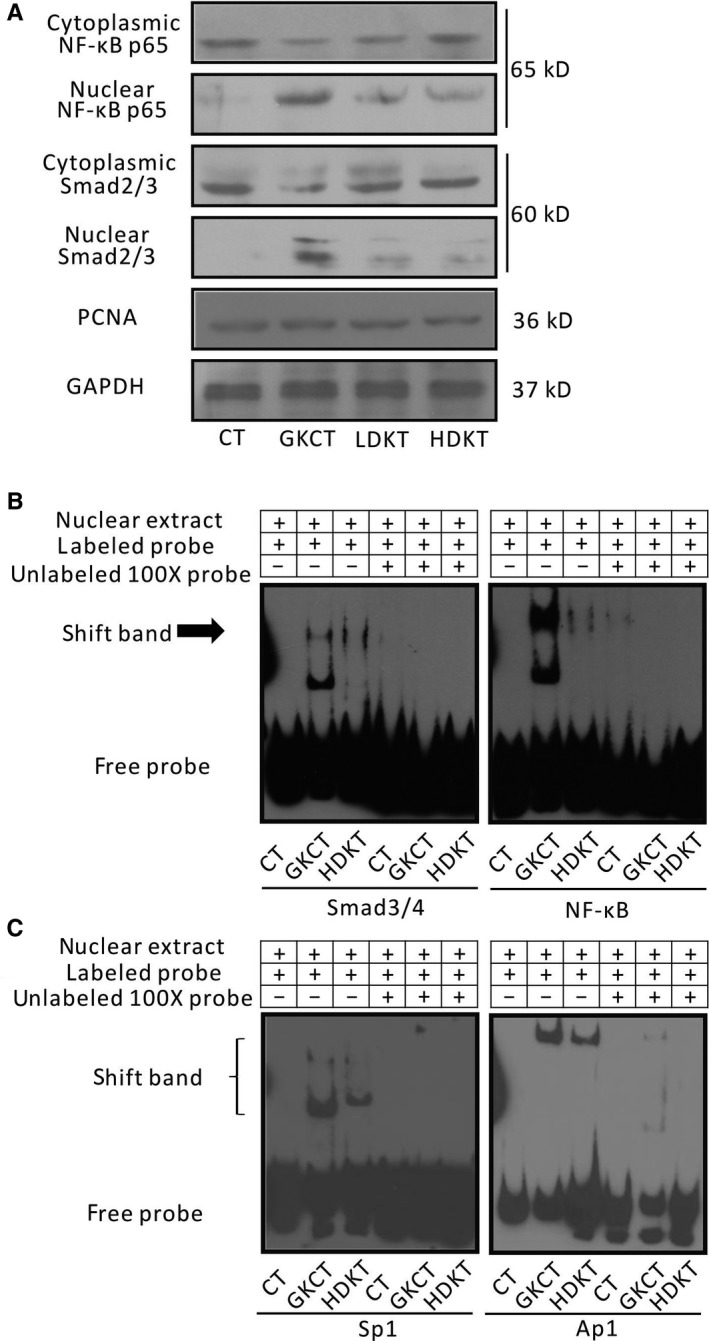
Kirenol inhibited NF‐κB and Smad signal pathways by Western blot and EMSA in heart of the GK rats. A, Cytoplasmic and nuclear extracts from the LV tissue of each group were tested for NF‐κB and Smad2/3 protein expression by Western blotting, B, Using EMSA detected the DNA binding activity of NF‐κB and Smad3/4, as well as Sp‐1 and AP1 in the nucleus of heart of the rats

The cytosolic and nuclear protein fractions from diabetic and non‐diabetic hearts were evaluated for inflammation and fibrosis‐related transcription factors including NF‐κB, Smads, Sp‐1 and AP1 by Western blotting and EMSA, respectively. As indicated in Figure 6A, Western blotting showed that kirenol administration not only attenuated the nuclear translocation of Smad 2/3 but also prevented the nuclear translocation of NF‐κB in a dose‐dependent manner. Furthermore, to investigate the contribution of NF‐κB and Smad 3/4 probes and Sp‐1 and AP1 probes binding to nuclear protein fractions, an EMSA was performed. The EMSA results revealed that the binding activity of the NF‐κB and Smad 3/4 probes and that of the Sp‐1 and AP1 probes to nuclear protein fractions were markedly attenuated after kirenol treatment (Figure 6B).

## 4 DISCUSSION

HG stimulates CF proliferation accompanied by the accumulation of both collagen I and III, characterized by α‐SMA expression and extracellular matrix protein expression, and TGF‐β1 expression (a pro‐fibrotic cytokine).^20^, ^21^, ^22^, ^23^ The results of the in vitro experiment in this study indicated that supplementation with kirenol prevented HG‐induced CFs proliferation by down‐regulating collagen I and III expression in CFs, decreasing secretion levels of collagen I in the supernatant, and decreasing α‐SMA expression in CFs in the presence of HG. Our present study also revealed that kirenol intervention decreased HG‐induced production of both TGF‐β1 and fibronectin in CFs and the expression of TGF‐β1 accumulated in the supernatant in a concentration‐dependent manner. A previous study revealed that HG induced multiple detrimental events including cytotoxicity, apoptosis and mitochondrial dysfunction in H9c2, causing a decrease in cell viability, an increased percentage of apoptotic cells and a loss of MMP.^24^ Accumulating evidence indicates that diabetes‐induced apoptosis often occurs accompanied by mitochondrial dysfunction,^25^ caspase‐3 signalling activation and down‐regulation of Bcl‐2 protein expression.^26^, ^27^, ^28^, ^29^ There were several important findings in our present work that suggested that kirenol interventions may reduce the rate of CMs apoptosis and re‐establish MMP in a concentration‐dependent manner in the presence of HG. We also found that kirenol concentration‐dependently decreased the ratio of Bax/Bcl‐2 and down‐regulated the expression level of cleaved caspase‐3. Considering that kirenol treatment has obvious in vitro effects of inhibiting CFs proliferation and collagen production and promoting anti‐apoptosis in the presence of HG, it is necessary to assess the effects of long‐term kirenol gavage on T2DM and associated remodelling in myocardium.

Various clinical studies have demonstrated that there is a close relationship between poor control of hyperglycaemia, hyperinsulinemia and diabetic complications, such as DCM.^7^, ^30^, ^31^, ^32^ Although kirenol gavage with either a 0.5 mg/kg daily dosage or 2.0 mg/kg daily dosage for 8 weeks did not affect bodyweight or lipid profiles in all groups of GK rats, oral administration of kirenol with a daily dose of 2.0 mg/kg decreased FPG levels and fasting plasma insulin by 22‐24 weeks of age in GK rats. Additionally, at the end of the observation period, HbA1c levels were decreased by kirenol administration only in the HDKT group.

Accumulating evidence from epidemiological studies has demonstrated that circulating levels of pro‐inflammatory cytokines, including TNF‐α and IL‐6, are positively correlated with the incidence of heart failure and are independent predictors of heart failure.^33^, ^34^ In the present study, we found that kirenol gavage for 8 weeks dose‐dependently decreased serum levels of TNF‐α, IL‐6 and IL‐1β in GK rats.

Currently, an increasing number of studies have demonstrated that several active ingredients extracted from different kinds of herbs that were once used as medicines for the treatment of inflammatory diseases, including arthritis, rheumatoid arthritis, nephritis and systemic lupus erythematosus, can be used to treat DCM and thereby improve cardiac function and weaken the structural abnormalities induced by diabetes in vivo and in vitro.^35^, ^36^, ^37^, ^38^ Furthermore, kirenol, which is derived from *Herba Siegesbeckiae*, is also a natural bioactive compound that has anti‐inflammatory properties and potent anti‐obesity activity in vitro and in vivo.^11^, ^15^, ^39^ In this study, for the first time, we found that kirenol administration for 8 weeks prevented a series of myocardial remodelling abnormalities, including cardiac dysfunction, hypertrophy, fibrosis and apoptosis, reduced inflammation, enhanced insulin signalling and decreased fibrotic signalling in GK rats. In addition, we observed that the effect of kirenol for preventing myocardial remodelling in GK rats was not correlated with decreases in blood glucose and plasma insulin. More importantly, since diabetes‐induced remodelling in cardiac tissue involves several abnormalities, including hypertrophy, fibrosis and apoptosis, that occur via modulation of multiple inflammatory signalling pathways that mainly converge towards both NF‐κB and MAPK signalling, inactivation of NF‐κB and MAPK signalling pathways using various bioactive components of herbal extracts may be a potential therapeutic approach, which has been investigated and confirmed by some preclinical studies.^9^, ^10^, ^40^, ^41^ Our present work for the first time showed that the obvious effects of kirenol on diabetic myocardial remodelling were due to decreased phosphorylation of MAPK signalling, including ERK1/2, JNK and p38MAPK, and inhibition of NF‐κB nuclear translocation in the LV tissue of GK rats, which may to be significant in the clinic.

The p65/50 subunits are the most abundant form of the NF‐κB family, which are inactive and bound to IκB in the cytoplasm of resting cells.^42^ Once IκB is phosphorylated after diabetic stimuli, the released p65/50 heterodimer then translocates to the nucleus and binds to its target gene promoter.^43^, ^44^ Our results showed that kirenol administration efficiently reduced diabetes‐induced phosphorylation of IκBα expression and inhibited the nuclear translocation of the NF‐κB p65 subunit under diabetic stimuli. Furthermore, observing alterations in the EMSA, administration of kirenol at a high dose attenuated NF‐κB transcriptional activity in the diabetic myocardium of GK rats.

Once DCM is established, cardiac insulin metabolic signalling is impaired in addition to reduced Akt phosphorylation.^7^, ^45^ Our data showed that kirenol treatment at high doses could not only reduce high insulin levels in circulation but also promote phosphorylated Akt activation in the diabetic myocardium of GK rats without intraperitoneal injection of insulin, while GK rats that did not receive kirenol gavage exhibited an obvious defect in response to insulin exposure.

**Figure 7:**
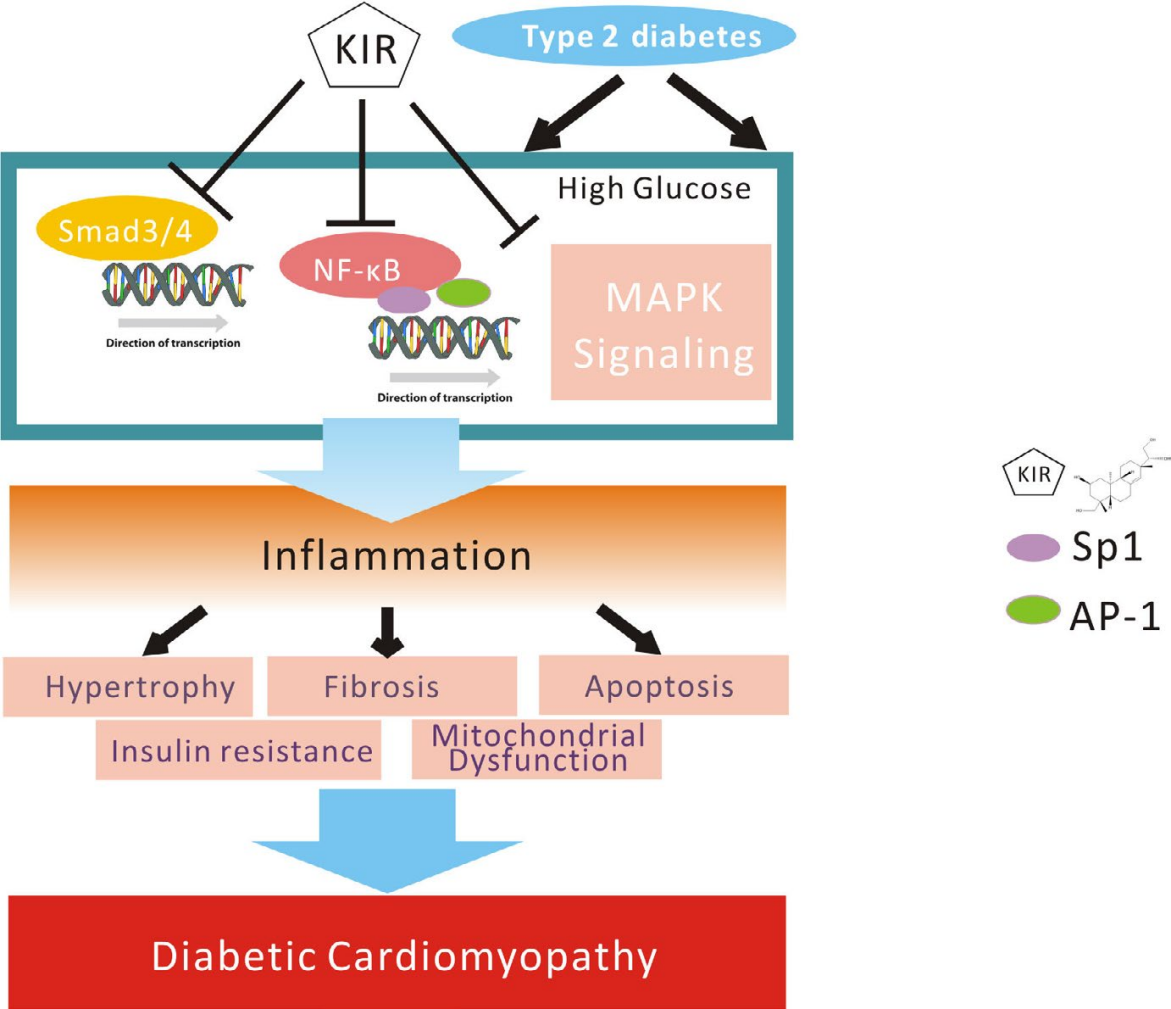
A schematic diagram for the kirenol treatment of diabetic cardiomyopathy in vitro and in vivo

Continuous Smad2/3 nuclear translocation may occur in response to stimulation from TGF‐β1 signalling; Smad2/3 then acts as a transcriptional factor in conjunction with Sp1 and exerts its effects on promoting fibrotic gene expression, including collagen I.^46^, ^47^ In addition, both ERK1/2 and p38 MAPK, members of MAPK subfamilies, also participate in regulating AP‐1 activation after TGF‐β stimulation in vitro.^48^, ^49^ In the present study, our data showed that kirenol gavage at a daily dosage of 2.0 mg/kg (high dose) inhibited the nuclear translocation of Smad2/3 and reduced the binding activities of Smad3/4 and the transcriptional coactivators Sp1 and AP‐1. Thus, these mechanisms suggest that kirenol treatment probably decreases pro‐fibrotic gene expression by inhibiting the activities of transcriptional factors including Smad3/4, Sp1 and AP‐1. Meanwhile, multiple potential effect and signalling pathways of kirenol treatment on DCM were summarized in Figure 7.

## 5 CONCLUSION

This study clearly shows that kirenol affects and collagen synthesis and attenuating ECM components and TGF‐β1 expression in vitro and in vivo. Moreover, kirenol treatment appears to have a significant anti‐apoptotic effect in vitro and in vivo. Our study for the first time demonstrates that the cardioprotective effect of kirenol in GK rats is independent of lowering HG and hyperinsulinemia and altering lipid profiles and occurs probably by regulating the NF‐κB, MAPK and TGF‐β/Smad signal pathways. Our study provides evidence that kirenol, as a bioactive component extracted from natural herbs, may have multiple effects that prevent remodelling that occurs during DCM in a T2DM model.

## CONFLICT OF INTEREST

The authors have no conflicts of interest to declare.

## AUTHOR CONTRIBUTIONS

B. Wu, X. Y. Huang and S. Wang contributed to the experimental design, data collection and analysis, and manuscript writing; L. Li, X. H. Fan, P. C. Li and C. Q. Huang contributed to data collection and analysis; J. Xiao contributed to interpretation of data; R. Gui contributed to study design and manuscript writing.

## Data Availability

All data generated or analysed during this study are included in this article.
