# Supplementary material for: Attenuation of diabetic cardiomyopathy by relying on kirenol to suppress inflammation in a diabetic rat model
Source: J Cell Mol Med. 2019 Sep 29;23(11):7651–63. doi: 10.1111/jcmm.14638 (PMC6815847; doi:10.1111/jcmm.14638)
Supplement: Supplementary file 1 [file JCMM-23-7651-s001.docx]

**Supplementary Figure 1.** The experimental procedure of the present study.


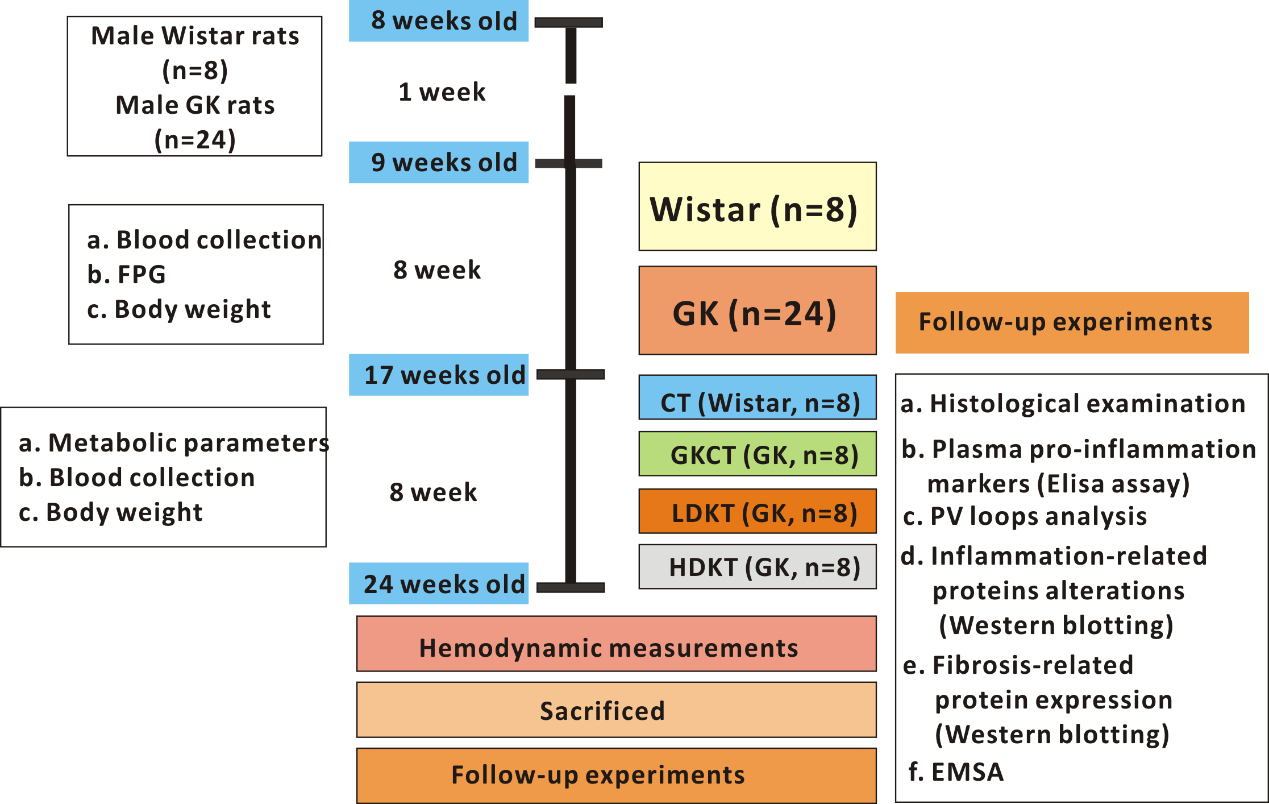


**Supplementary Figure 2.** Alterations in body weight, FPG and fasting plasma insulin of all groups over 8 weeks of kirenol gavage. (A) body weight, (B) FPG and (C) fasting plasma insulin. ^*^*P*<0.01


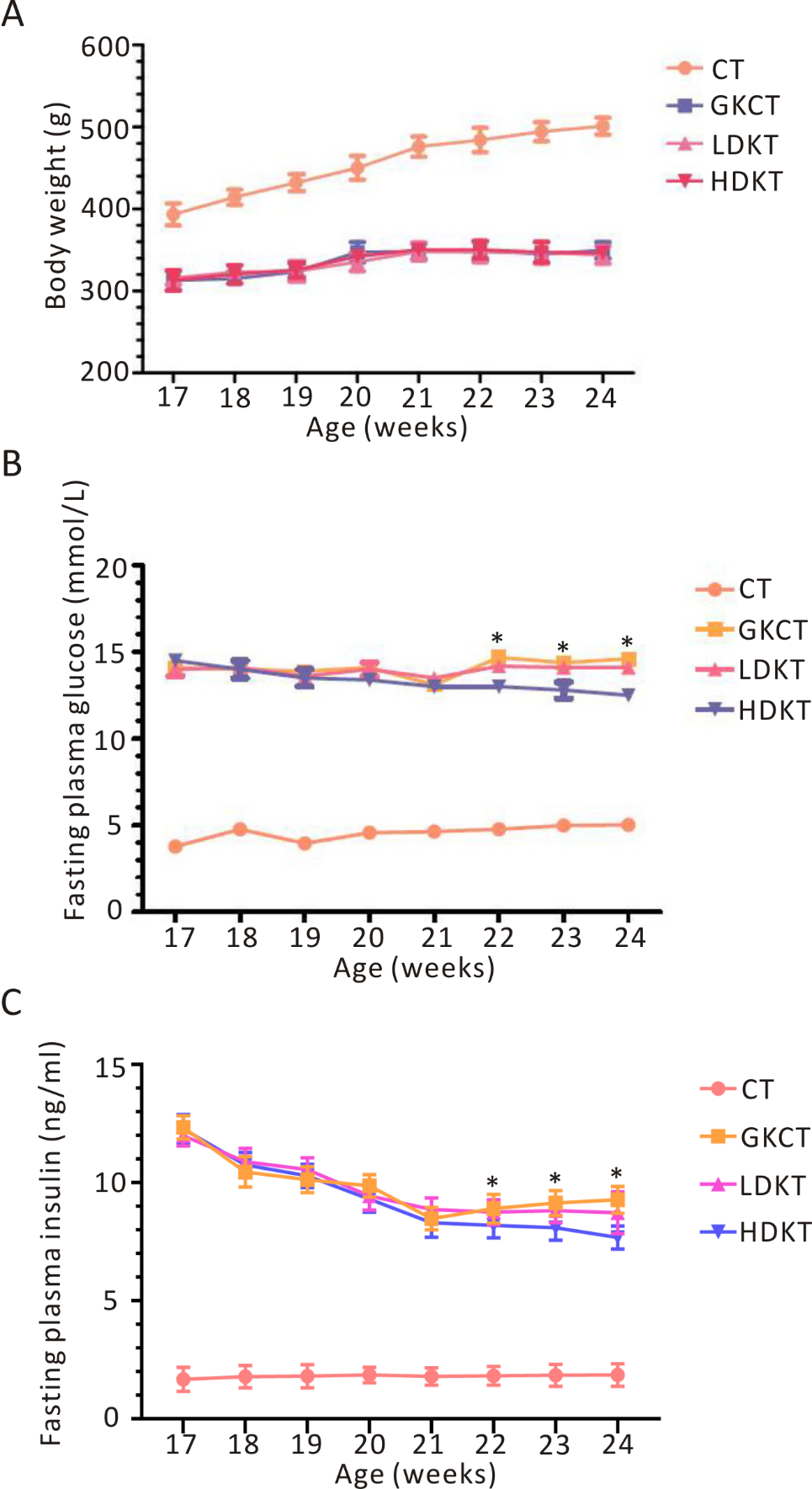


**Supplementary Figure 3.** Effect of kirenol on the hypertrophic and fibrotic markers protein expression in heart of the rats. (A) The protein expression levels of ANP and BNP from each group detected by western blotting, (B) Diabetes-induced protein expression of fibrotic markers including type I collagen, type III collagen, fibronectin, α-SMA, CTGF and TGF-β1 was determined by western blotting. ^*^*P*<0.01 vs. CT; ^**^*P*<0.01, ^#^*P*<0.01 vs. GKCT, ^***^*P*<0.01 vs. LDKT


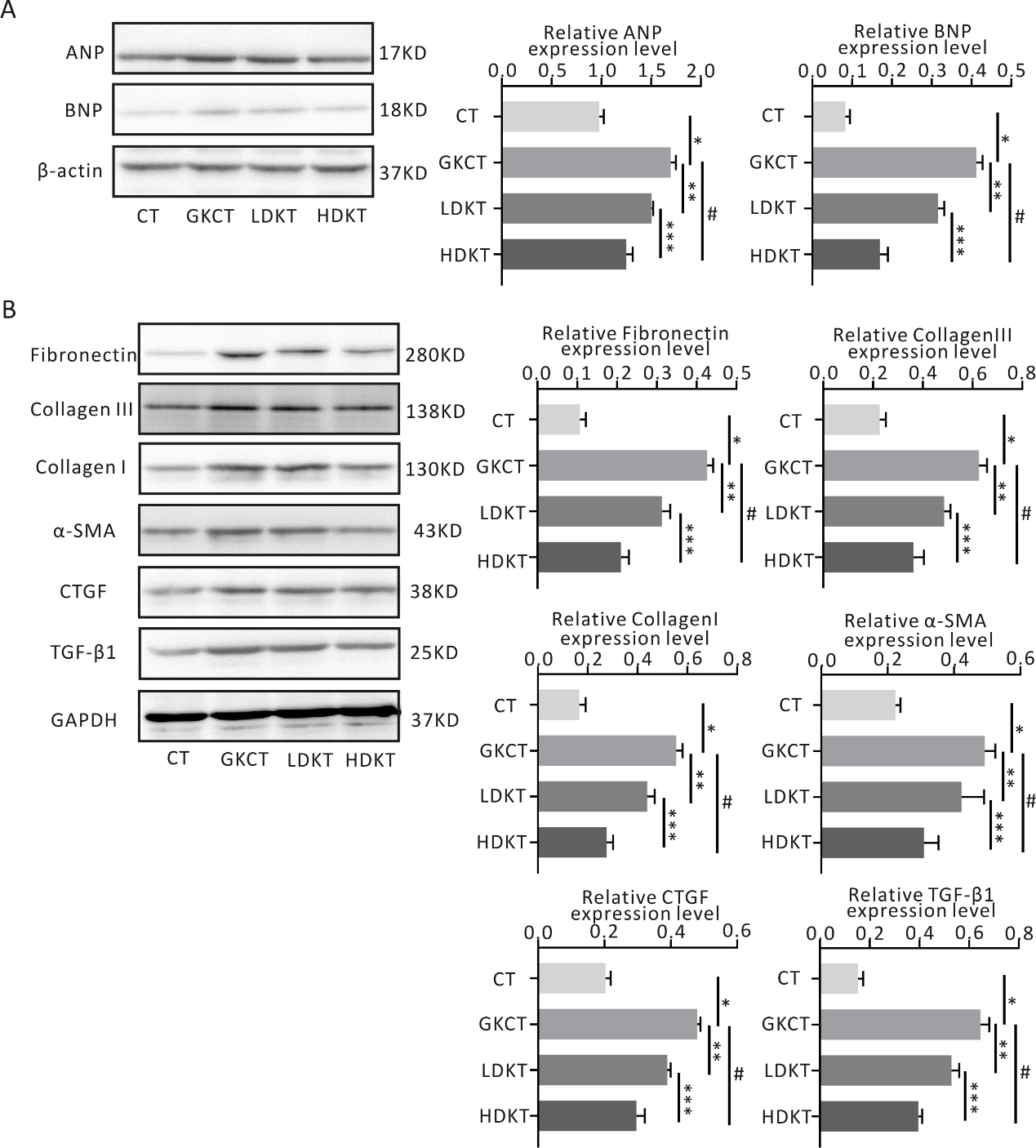


**Supplementary Figure 4.** Kirenol enhanced Akt phosphorylation in diabetic heart of the GK rats. Western blotting analysis of the experimental animals with or without treatment of kirenol, then determined for basal or insulin-induced phosphorylation expression. ^*^*P*<0.01 vs. CT+Insulin; ^**^*P*<0.01, ^***^*P*<0.01 vs. GKCT; ^#^*P*<0.01, ^@^*P*<0.01 vs. GKCT+Insulin, ^NS^*P*>0.05 vs. CT+Insulin


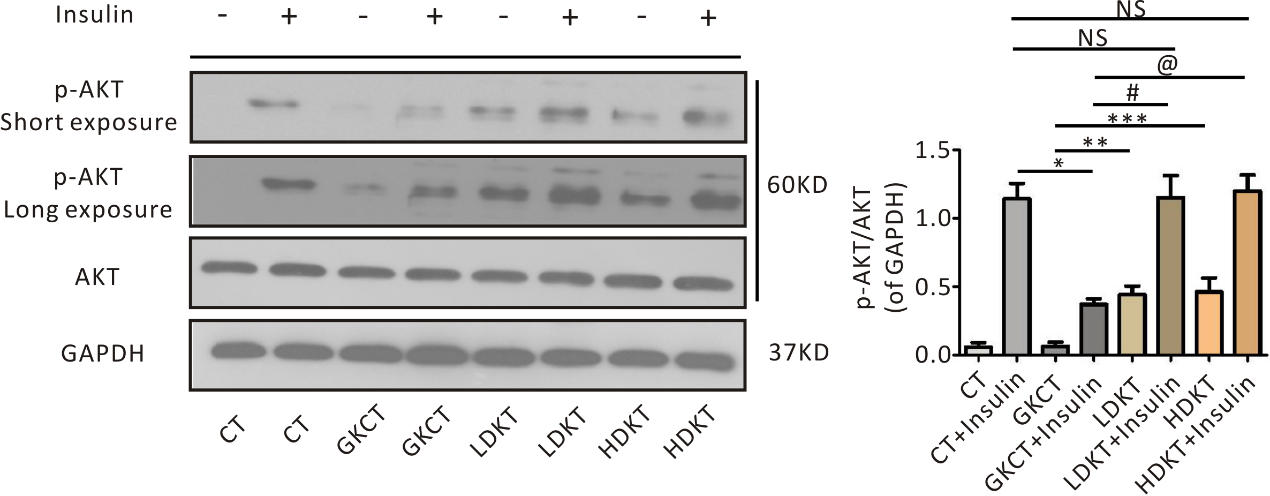


| **Table S1 Sequence of EMSA probes** | |
| --- | --- |
| TF | Probes |
| NF-κB | 5'-AGTTGAGGGGACTTTCCCAGGC-3' |
|  | 3'-TCAACTCCCCTGAAAGGGTCCG-5' |
| SP1 | 5'-ATTTCGATCGGGGCGGGGCGAGC-3' |
|  | 3'-TAAGCTAGCCCCGCCCCGCTCG-5' |
| AP1 | 5'-CGCTTGATGACTCAGCCGGAA-3' |
|  | 3'-GCGAACTACTGAGTCGGCCTT-5' |
| Smad3/4 | 5'-AGTATGTCTAGACTGA-3' |
|  | 3'-TCATACAGATCTGACT-5' |

| **TableS2 Blood biochemical parameters of experimental rats** | | | | |
| --- | --- | --- | --- | --- |
| Parameters | CT (Wistar) (n=8) | GKCT (n=8) | LDKT(n=8) | HDKT (n=8) |
| HbA1c (%) | 4.32±0.33 | 9.63±0.66 | 9.59±0.58 | 9.39±0.55^*^ |
| Triglycerides (mmol/L) | 0.93±0.19 | 3.78±0.75 | 3.75±0.63 | 3.73±0.58 |
| Total cholesterol (mmol/L) | 2.11±0.31 | 2.57±0.41 | 2.56±0.45 | 2.55±0.40 |
| HDL-Cholesterol (mmol/L) | 0.23±0.06 | 2.21±0.16 | 2.20±0.13 | 2.20±0.16 |
| LDL-Cholesterol (mmol/L) | 0.76±0.22 | 1.08±0.20 | 1.07±0.20 | 1.07±0.17 |
| LDL-cholesterol: low density lipoprotein-cholesterol, HDL-cholesterol: high density lipoprotein-cholesterol. ^*^*P*<0.01 vs. GKCT | | | | |
|  |  |  |  |  |

| **TableS3 Comparison of mean plasma levels of pro-inflammatory cytokines in all groups at the end of observations** | | | | |
| --- | --- | --- | --- | --- |
|  |  |  |  |  |
| Group | CT | GKCT | LDKT | HDKT |
| TNF-α(pg/ml) | 94.63±7.21 | 147.81±9.92^*^ | 122.43±12.17^**^ | 110.26±7.38^#@^ |
| IL-6 (pg/ml) | 61.51±6.09 | 108.76±11.09^*^ | 93.40±9.31^**^ | 83.44±9.89^#@^ |
| IL-1β (pg/ml) | 82.19±15.75 | 160.71±18.17^*^ | 148.03±19.62^**^ | 123.27±13.42^#@^ |
| Values are presented as means ± S.D., ^*^*P*<0.01 vs. CT, ^**^*P*<0.01, ^#^*P*<0.01 vs. GKCT, ^@^*P*<0.01 vs. LDKT | | | | |

| **TableS4 Hemodynamic parameters of the study groups** | | | | |
| --- | --- | --- | --- | --- |
| Variable | CT (n=8) | GKCT (n=8) | LDKT (n=8) | HDKT (n=8) |
| HR (bpm) | 521±23 | 357±15^*^ | 386±17^**^ | 436±21^#@^ |
| Pes (mmHg) | 177.1±11.8 | 137.1±25.2^*^ | 155.4±9.3^**^ | 165.3±14.8^#@^ |
| Ped (mmHg) | 2.1±1.3 | 5.9±2.6^*^ | 4.7±2.1^**^ | 3.9±4.4^#@^ |
| dp/dt_max_ (mmHg/s) | 14676±2436 | 10311±3052^*^ | 11201±399^**^ | 12428±799^#@^ |
| dp/dt_min_ (mmHg/s) | -15795±1647 | -10146±2578^*^ | -11451±514^**^ | -12143±804^#@^ |
| HR: heart rate, Pes: LV end systolic pressure, Ped: LV end diastolic pressure, dp/dt_max_: maximal rates of rise of ventricular pressure, dp/dt_min_: maximal rates of decline of ventricular pressure. Values are presented as means ± S.D., ^*^*P*<0.01 vs. CT, ^**^*P*<0.01, ^#^*P*<0.01 vs. GKCT, ^@^*P*<0.01 vs. LDKT | | | | |
|  |  |  |  |  |
|  |  |  |  |  |
|  |  |  |  |  |
|  |  |  |  |  |
